# Functional Integration between Salience and Central Executive Networks: A Role for Action Video Game Experience

**DOI:** 10.1155/2016/9803165

**Published:** 2016-01-14

**Authors:** Diankun Gong, Hui He, Weiyi Ma, Dongbo Liu, Mengting Huang, Li Dong, Jinnan Gong, Jianfu Li, Cheng Luo, Dezhong Yao

**Affiliations:** ^1^Key Laboratory for NeuroInformation of Ministry of Education, School of Life Science and Technology, University of Electronic Science and Technology of China, Chengdu 610054, China; ^2^ARC Centre of Excellence in Cognition and Its Disorders, Macquarie University, Sydney 2109, NSW, Australia

## Abstract

Action video games (AVGs) have attracted increasing research attention as they offer a unique perspective into the relation between active learning and neural plasticity. However, little research has examined the relation between AVG experience and the plasticity of neural network mechanisms. It has been proposed that AVG experience is related to the integration between Salience Network (SN) and Central Executive Network (CEN), which are responsible for attention and working memory, respectively, two cognitive functions essential for AVG playing. This study initiated a systematic investigation of this proposition by analyzing AVG experts' and amateurs' resting-state brain functions through graph theoretical analyses and functional connectivity. Results reveal enhanced intra- and internetwork functional integrations in AVG experts compared to amateurs. The findings support the possible relation between AVG experience and the neural network plasticity.

## 1. Introduction

One of the most prominent changes to our modern lives is the use of computers, which has adapted our entertainment experience with the introduction of video games. The action video game (AVG) is major video game genre that offers an important virtual environment for human behaviors and has increased exponentially in popularity worldwide over a wide age range. Similar to conventional sports (e.g., basketball and football), AVG is physically and mentally demanding as it requires multiple cognitive functions including hand-eye coordination, working memory, and attention (see Footnote for an example of AVG) [1]. Furthermore, AVG influences cognitive development adaptively, thus offering an important venue to examine the relation between active learning and neural plasticity [2].

Research has shown that AVG experience is related to enhanced primary (e.g., visual processing [3–5], eye-hand coordination [6], contrast sensitivity [4], oculomotor performance [7], and body movement [8]) and higher-level cognitive functions (e.g., attention and working memory). For example, AVG experts tend to have better selective attention than amateurs, and AVG training improves attentional performance in the amateurs, thus supporting the attentional effects of AVG experience. Research also shows that AVG experts have enhanced spatial distribution of visuospatial attention [9], attentional capture [10], and attention shifting at switching tasks [11]. In addition, AVG experts tend to have better visual short-term and working memory than amateurs, and AVG training enhances visual working memory in amateurs [12–14]. For example, Colzato et al. [12] found that AVG experts were faster and more accurate in *n*-back task than amateurs. Furthermore, Blacker et al. [13] examined whether AVG training could increase the quantity and/or the quality of information stored in visual working memory (VWM). Results revealed a significant increase in the VWM capacity after the training, as measured by a change detection task.

Research has also examined the neural basis of the cognitive benefits of AVG experience. A recent study shows that AVG experts have enhanced functional connectivity and grey matter volume in insular subregions compared to amateurs [15]. AVG experts also have better early filtering of irrelevant information and selective attention than amateurs, as measured by neural activities in frontoparietal areas [16]. Furthermore, AVG experience is related to gray matter volume (GMV) in brain areas responsible for attention and working memory (e.g., dorsal striatum [17], right posterior parietal area [18], entorhinal area, hippocampal gyrus, occipital lobe [19], and dorsolateral prefrontal cortex [20]). In addition, AVG training improved older adults' cognitive control by reducing the multitasking cost as measured by electrophysiological signatures [21].

However, little research has examined the relation between AVG experience and the integration of attentional and working memory networks. Research of resting-state functional connectivity (FC, a dynamic coordinated activity for communicating information on connected brain regions [22, 23]) reveals two separate functional networks for attention and working memory, respectively [24–29]. The Salience Network (SN), typically including anterior cingulate cortex (ACC) and anterior insula, supports the detection of salient events. The Central Executive Network (CEN), typically including the dorsolateral prefrontal cortex (DLPFC) and posterior parietal cortex (PPC), supports attentional control and working memory (see Table 2 for nodal information of the networks).

SN and CEN may interact with each other in supporting attention and working memory [27, 30]. Specifically, SN receives and provides selective amplification of salient information thereafter generates a top-down control signal that initiates CEN to respond to salient information for attentional shift and control execution [31]. Thus, research has proposed that enhanced attention and working memory improve the functional integration of SN and CEN [32] and that this improvement may be the neural basis of expert attention and working memory [27]. AVG experience therefore offers an important venue to test this proposition, since AVG requires a high level of attention and working memory. This study examines the relation between AVG experience and the integration of SN and CEN by comparing AVG experts and amateurs. We first analyzed functional integration using the graph-theoretical analysis and then located the functional integration using the FC analysis. Graph theory is the study of graphs, which are mathematical structures used to model pairwise relations between objects. A graph is a set of nodes (vertices) linked by connections (edges) and provides an abstract representation of the elements and their interactions in a system. Graph theory has been widely used to quantitatively characterize topological organization of functional networks. We predict that if AVG experience is related to the integration of SN and CEN, experts should have enhanced functional integration compared to amateurs.

## 2. Materials and Methods

### 2.1. Participants

Participants gave written consent to participate in this study, which was approved (20150035) by the Ethics Board of the University of Electronic Science and Technology of China (UESTC). Twenty-three AVG experts (M = 23.3 yrs ± 4.3) and 22 amateurs (M = 22.3 yrs ± 3.46) participated in this study. The experts were experienced AVG players (i.e., League of Legends (LOL) or Defense of the Ancient (DOTA)). Based on a preliminary self-report questionnaire, the experts had at least four years of AVG experience, while the amateurs had less than one year of AVG experience. AVG expertise was quantified based on the Elo rating generated by the AVG program [33]. The experts' score ranged from 1800 to 2600 points, while the amateurs' score was below 1200, verifying the group membership. The Elo rating scale is widely used as a predictor of the outcome of a multiplayer AVG game. A 100-point advantage indicates a 64% probability of winning a game, while a 200-point advantage indicates a 76% probability.

The two groups were matched in years of school education, IQ as measured by Raven's Progressive Matrices (experts: 91 ± 10.8* versus* amateurs: 91.6 ± 9.8) prior to this study, and the onset age (8 years) of video game playing (not necessarily AVG). All the participants were male and right-handed based on the Edinburgh Inventory [34], reported normal or corrected-to-normal vision, and presented no history of neurological illnesses.

To allow for the examination of the relation between behavioral and fMRI data, participants completed a digital *n*-back task and a spatial memory task before an fMRI session. In the digital *n*-back test, they first saw a sequence of digits and were then asked to indicate whether a digit matched the one from *n* steps earlier in the sequence. The task difficulty was adjustable by the load factor – *n*, ranging from 0 to 2 [35]. In the spatial memory task, participants first saw a sequence of blocks being lit up within a 6 × 6 grid consisting of 36 blocks and were asked to memorize the sequence and then repeat it. Starting with a short sequence which increased to a 15-block sequence maximally, this task measured the longest sequence one could remember [36]. These tasks were used because (1) they require both spatial attention and working memory that are essential for AVG playing and (2) the task difficulty is dynamically adjustable based on participants' performance, making these tasks sensitive indicators to one's spatial attention and working memory in a computer game session. Thus, although the tasks are not standardized tests of spatial attention and working memory, they offered sufficiently sensitive behavioral data for us to examine their relation to fMRI data.

### 2.2. Data Acquisition

Images were collected on a 3T MRI scanner (GE Discovery MR750) at the UESTC MRI Research Center. Resting-state fMRI data were acquired using gradient-echo EPI sequences (repetition time (TR) = 2000 msec, echo time (TE) = 30 msec, flap angle (FA) = 90°, matrix = 64 × 64, field of view (FOV) = 24 × 24 cm^2^, and slice thickness/gap = 4 mm/0.4 mm), with an eight-channel-phased array head coil. All the participants underwent a 510-second resting-state scanning to yield 255 volumes (32 slices per volume). High-resolution T1-weighted images were acquired using a 3-dimensional fast spoiled gradient echo (T1-3D FSPGR) sequence (TR = 6.008 msec, TE = 1.984 msec, FA = 9°, matrix = 256 × 256, FOV = 25.6 × 20 cm^2^ (80%), and slice thickness (no gap) = 1 mm) to generate 152 slices.

### 2.3. fMRI Data Preprocessing

The fMRI data were processed through typical preprocessing procedures using SPM8 software (Wellcome Department of Cognitive Neurology, London, UK) [37, 38], including the first five volumes of each run discarded, slice scan time correction, head motion correction [37], normalized images with a BOLD EPI template in the Montreal Neurological Institute (MNI) atlas space, and spatial smoothing with Gaussian kernel of 8 mm full-width half-maximum (FWHM). Temporal filtering (band-pass) was then performed between 0.01 and 0.08 Hz. BOLD time courses were extracted from each ROI by averaging 27 voxels. The linear regression was used to reduce the effects of physiological processes (e.g., the fluctuations of cardiac and respiratory cycles). Otherwise, the 9 noise covariates were added in the regression analysis, including White Matter (WM), Cerebro-Spinal Fluid (CSF), Global Signal (GS), as well as 6 motion parameters (3 rotations and 3 translations as saved by the 3D motion correction) [39–42]. We derived the GS/WM/CSF nuisance signals averaging the time courses of the voxels in each subject's whole brain/WM/CSF masks. To derive these masks, coregistration between T1 and functional image as well as the segmentation process of each participant's T1 image were performed.

### 2.4. Functional Network Analysis

The quantitative metrics of SN and CEN were analyzed based on the graph-theoretical method for the full correlation matrix using the Brain Connectivity Toolbox (http://www.brain-connectivity-toolbox.net/) [43]. The topological properties of the functional networks were defined on the basis of a 23 × 23 binary graphs, *G*, consisting of nodes (each ROI) and undirected edges (functional connectivity) between nodes. This binary graph was constructed by applying a correlation threshold *T* to the correlation coefficients. Consider

(1) $$\begin{array}{c} e_{ij}=\left\{\begin{array}{cc} 1, & \text{If }r_{i,j}T,\\ 0, & \text{otherwise,} \end{array}\right. \end{array}$$

where *e*_*ij*_ refers to the edge in the graph. When *r*_*ij*_ of a pair of nodes, *i* and *j*, exceeds a given threshold *T*, an edge is assumed to exist; it does not exist otherwise. Since there is no gold standard defining the threshold *T* based on the literature, we used a variety of thresholds ranging from 0.05 to 0.3 in 25 steps. Since all the thresholds generated a similar pattern of results, the threshold of 0.2 was used for the data report.

We then conducted the graph-theoretical analysis. At the network level, we analyzed three characteristics (i.e., global efficiency, cost, and mean clustering coefficient). Three nodal characteristics were also analyzed (i.e., nodal clustering coefficient, nodal degree, and nodal efficiency) [43]. The nodal degree was defined by the number of links connected to the node, which was equivalent to the number of neighbors that the node had. See Table 1 for the mathematical formulas used in graph-theoretical analyses (see [44] for details).

The between-group comparisons were conducted using the nonparametric permutation test, a method widely used when normality assumption was violated [45, 46]. For a given parameter, we first estimated *t* value to indicate the between-group difference. We then randomly assigned the parameter values of all the participants to two groups to recalculate *t* value between the two randomized groups. We repeated the permutation 10,000 times and obtained 10,000 *t* values. Finally, we determined the significance level of the between-group differences at 95% of the empirical distribution in a two-tailed test [47].

ROIs and the network construction were defined based on the methods and findings of previous research [24, 25, 38, 48, 49]. The network construction of SN was defined according to Seeley et al.'s method [25]: we first selected anterior insula as the start ROI of SN; we then computed the FC between anterior insula and other brain areas; multiple comparison corrections were then conducted with FDR *p* < 0.05, and the survival clusters were the other ROIs of SN. This analysis showed that, in addition to ACC, survival clusters also included SMG, MFG, and posterior insula. This is consistent with Cauda et al.'s findings [38] (see Cauda et al.'s supplementary materials for details). The ROIs of CEN were defined according to Markett et al.'s [49] and Spreng et al.'s findings [48].

Thus, we selected 23 MNI coordinates as the center positions of functional network nodes (ROIs) (Table 2). Functional network edges were defined by Pearson's correlation coefficients computed between the extracted signals of ROIs for each participant. The correlation coefficients then underwent Fisher's *r*-to-*z* transformation [50]. For each edge, independent samples *t*-tests analyzed the between-groups difference and corrected the multiple comparisons with False Discovery Rate (FDR, *p* < 0.05). To analyze the relation between SN and CEN, we calculated the average nodal signal across all the nodes within SN and CEN, respectively.

## 3. Results

### 3.1. Increased Global Characteristics

**Figure 1:**
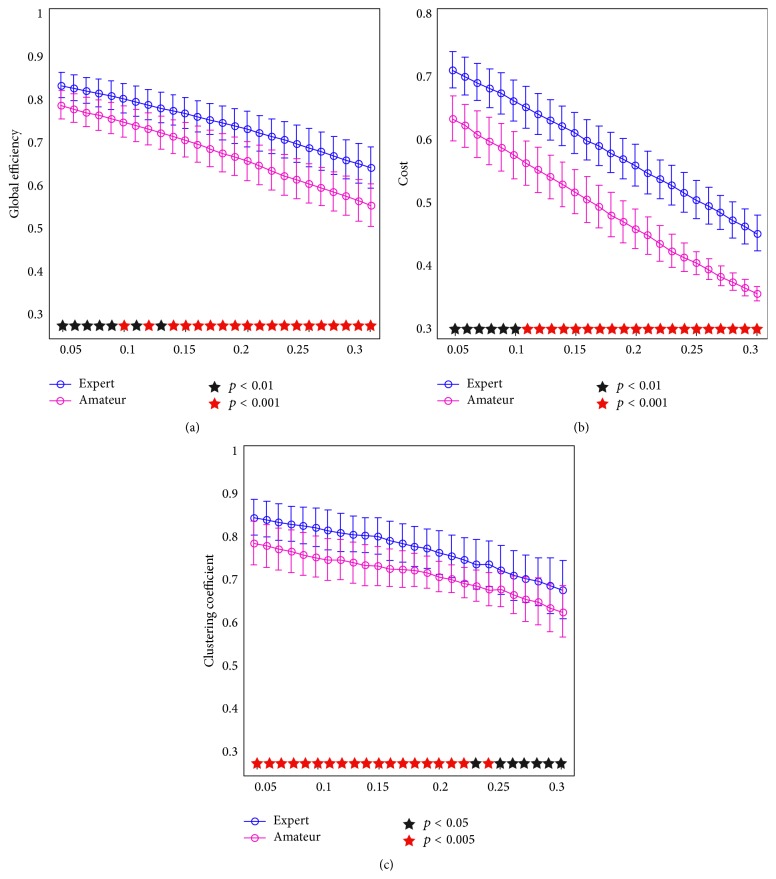
Increased global characteristics in AVG experts over amateurs. (a), (b), and (c) indicate global efficiency, connection cost, and mean clustering coefficient, respectively. The abscissa indicated step-by-step thresholds (correlation coefficient) to establish a network.

For quantitative metric of the integration between SN and CEN, we constituted the nodes of both SN and CEN into a multisystem network. At different threshold levels as shown in Figure 1, graph-theoretical analyses showed significant increases in the three global characteristics (i.e., global efficiency, connection cost, and the mean clustering coefficients in the multisystem network) in the experts compared to the amateurs (see Figure 1).

### 3.2. Increased Nodal Characteristics

**Figure 2:**
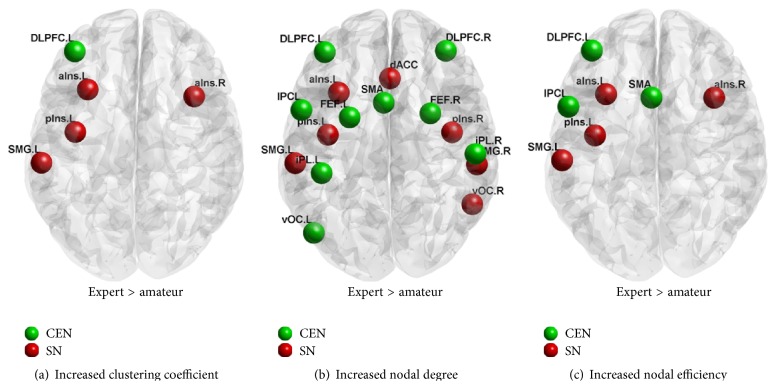
Increased nodal characteristics in the experts over the amateurs. (a), (b), and (c) indicate significantly increased nodal clustering coefficient, degree, and efficiency, respectively. Green dots are the nodes of CEN, while red dots are the nodes of SN.

In the new network, we found significantly enhanced nodal characteristics in the experts compared to the amateurs (Figure 2).  Figure 2(a) showed that DLPFC.L of CEN and four nodes of SN had increased nodal clustering coefficient (bilateral alns, plns.L, and SMG.L).  Figure 2(b) showed a significantly increased nodal degree in most of the SN and CEN nodes.  Figure 2(c) revealed a pattern of results similar to Figure 2(a) except IPCL and SMA of CEN in nodal efficiency.

### 3.3. Enhanced FC in the Intranetworks and Internetworks

**Figure 3:**
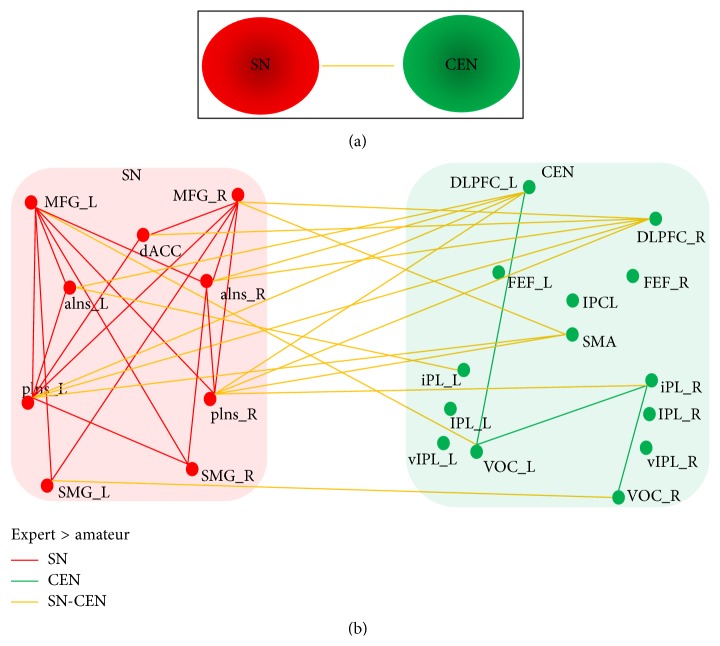
The significantly enhanced FC in the experts. (a) indicates enhanced FC between SN and CEN based on the correlational analysis between the average nodal signal of SN and CEN (yellow lines indicate *p* < 0.001). (b) indicates enhanced FC at the nodal level (FDR, *p* < 0.05). Red dots are the nodes of SN; red lines are the edges of SN; green dots are the nodes of CEN; green lines are the edges of CEN; yellow lines are edges of internetwork.

We examined the intranetwork FC through the correlation between nodes within each network (SN and CEN). The results showed that the experts had a significantly enhanced intranetwork FC within both SN and CEN. In Figure 3, red lines indicate enhanced edges within SN, while green lines indicate enhanced edges within CEN.

The average nodal signal was calculated across all the nodes within SN and CEN, respectively. Then, we examined the internetwork FC through the correlation between the average nodal signal of SN and CEN. The experts had a significantly enhanced internetwork FC between SN and CEN than the amateurs (experts: Mean = 0.82, SD = 0.21; amateurs: Mean = 0.51, SD = 0.32, *t* = 3.91, and *p* < 0.001, Figure 3(a)). The enhanced internetwork FC was mostly evident in bilateral DLPFC and SMA in CEN, while SN showed a more even spatial distribution since the majority of the nodes were related to the enhancement of internetwork FC. Furthermore, the experts did not have decreased FC compared to the amateurs.

### 3.4. Correlations between Behavioral Data and Graph-Theoretical Characteristics

The experts outperformed the amateurs in the spatial memory task (*t* = 4.07 and *p* < 0.001) and responded faster (but similarly accurately) in the 2-back task (*t* = −2.08 and *p* = 0.04). In addition, the experts' performance on the spatial memory task was positively related to the global efficiency (*r* = 0.47 and *p* = 0.04) and the connection cost (*r* = 0.48 and *p* = 0.03); furthermore, the response time in the 2-back task was negatively correlated to the nodal efficiency of DLPFC.L (*r* = −0.51 and *p* = 0.02). The same analyses in the amateurs did not reveal significant correlations between behavioral data and graph-theoretical characteristics.

## 4. Discussion

This study examined the functional integration of SN and CEN in AVG experts and amateurs. Results showed that experts had enhanced global characteristics, nodal characteristics, and FC both within and between networks compared to amateurs. Thus, AVG experience may be related to enhanced integration between SN and CEN.

### 4.1. Enhanced Global Characteristics

Research suggests that both SN and CEN are activated in certain cognitive tasks [27]. Using graph-theoretical analyses, we examined the multisystem network (SN and CEN combined) and found significantly enhanced global characteristics in AVG experts compared to amateurs, including global efficiency, mean clustering coefficient, and connections cost (Figure 1). Global characteristics often indicate the global information of a network. Specifically, global efficiency reflects the ability of a network to integrate all the nodal information; mean clustering coefficient indicates the nodal information processing of a network in the centralized tendency; connection cost denotes the resource consumption in maintaining the function of a network [43, 44]. Thus, the current findings suggested that the experts might be advanced in processing network information. Furthermore, these enhancements were realized with increased resource consumption in maintaining the function of networks, consistent with previous findings on the neural network [51]. The correlations between behavioral data (performance of the spatial memory) and global characteristics (global efficiency and connection cost) suggested that this enhanced efficiency of the global network might in turn improve the performance in an AVG session.

### 4.2. Enhanced Nodal Characteristics

We evaluated three nodal characteristics: clustering coefficient, degree, and efficiency. Nodal clustering coefficient indicates the ability of information processing of a node; nodal degree reflects the number of connections of a node, a basic nodal characteristic to which other nodal characteristics are related; nodal efficiency reflects the ability of a node to integrate specialized information from other nodes [43, 47]. Thus, the experts' increased nodal characteristics suggest that they have an enhanced information processing ability in local regions of CEN and SN. These regions are shown in Figure 2. It is noteworthy that the nodes with enhancements of all the three characteristics (i.e., DLPFC, Insula, and SMG) may be exceptionally closely related to AVG experience. Furthermore, we found that DLPFC.L, an important node in CEN, is related to attentional control and working memory [25, 27].

Consistent with a recent study that found an enhanced GMV in DLPFC in AVG experts, the present study showed an enhanced DLPFC.L [20]. Furthermore, nodal efficiency of DLPFC.L was correlated with the response time in the 2-back task in the experts but not in the amateurs. AVG experts also had better working memory than amateurs. Thus, the left DLPFC might play an exceptionally important role in the cognitive effects of AVG experience. Furthermore, the bilateral DLPFC, which are the main nodes in CEN, had greater enhanced FC between CEN and SN than other nodes, suggesting that DLPFC might be related to the integration of both CEN and SN.

In addition, bilateral alns receives salient information and initiating CEN. According to a recent resting-state study, bilateral plns and SMG.L are related to the sensorimotor network [38] which charge input and output information to support attentional and working memory. Thus, these enhanced nodal characteristics support the behavioral finding that AVG experience is related to advanced attention and working memory [12, 52, 53].

### 4.3. Enhanced Functional Integration

To further locate the functional integration, we examined intranetwork and internetwork using FC analyses. We found that the experts had significantly enhanced FC in the intranetworks compared to the amateurs (see Figure 3 (red and green lines)). Furthermore, the enhanced FC between the nodes of SN and CEN (e.g., dACC, iPL_L, bilateral AI, PI, DLPFC, and MFG) suggests an enhanced functional integration between SN and CEN, which may facilitate attention and working memory in a cognitively demanding task [25, 48]. Enhanced functional integration between SN and CEN observed in the experts might be the neural basis of expert attention and working memory in an AVG session [12, 52–54]. These enhancements observed at the global level in the present study are consistent with our recent findings that AVG experts have enhancements in insular subregions observed at the local level. These findings support the possible relation between AVG experience and enhancements of functional integration.

Nevertheless, the correlational nature of this study precludes causal inferences. For example, AVG experts may have an innate advanced attentional ability, which in turn may reinforce their interest in AVG. In addition, AVG experts may lead a more active life than amateurs, which may also contribute to the network change. Nevertheless, we found a possible relation between AVG experience and the integration of functional networks, motivating follow-up experimental studies to test the causal effect of AVG training on neural plasticity. An experimental study that is currently in progress shows preliminary evidence supporting the causal effect of AVG training on neural plasticity. In addition, future studies should recruit a control group that has no video game experience. This design can reveal any* qualitative* distinctions related to the AVG experience [55].

## 5. Conclusions

By comparing AVG experts with amateurs, this study found that AVG experts had significantly enhanced global characteristics, nodal characteristics, and FC in SN and CEN. Thus, long-term AVG playing is related to the integration between SN and CEN. The integration may be related to the experts' advanced attention and working memory in a game session.

## Figures and Tables

**Table 1:** Mathematical formulas used in graph theoretical analyses.

Global efficiency	Connection cost	Nodal efficiency	Nodal clustering coefficient
Eglobal=1N(N-1)∑i≠j∈G1Lij1Lij2	Kcost=1N(N-1)∑i∈GKi1Lij2	Ei=1(N-1)∑i≠j∈G1Lij1Lij2	$C_{i}=\frac{e_{i}}{K_{i}(K_{i}-1)/2}$

Note: we defined the subgraph *G*_*i*_ as the set of nodes which are the direct neighbors of the *i*th node, which is directly connected to the *i*th node with an edge. The degree of each node, *K*_*i*,*i*=1,2,…,23_, is defined as the number of nodes in the subgraph *G*_*i*_.

**Table 2:** The selected ROIs for data analysis (Number 1~9 ROIs = SN, 10~23 ROIs = CEN).

ROI number	Network	Abbrev.	Coordinate (MNI)			Brain area
1	SN	*aIns_L*	−31	21	−2	*Anterior insula*
2		*aIns_R*	39	19	−3	
3		*pIns_L*	−40	−4	2	*Posterior insula*
4		*pIns_R*	42	−6	0	
5		*dACC*	2	22	28	*Dorsal anterior cingulate cortex*
6		*MFG_L*	−37	42	25	*Middle frontal gyrus*
7		*MFG_R*	34	45	22	
8		*SMG_L*	−59	−35	29	*Supramarginal gyrus*
9		*SMG_R*	58	−37	33	

10	CEN	IPS_L	−23	−70	46	Left intraparietal sulcus
11		IPS_R	25	−62	53	
12		iPL_L	−42	−48	51	Inferior parietal lobule
13		iPL_R	57	−36	54	
14		vIPS_L	−15	−90	24	Ventral parietal sulcus
15		vIPS_R	35	−85	27	
16		FEF_L	−24	−15	66	Frontal eye field
17		FEF_R	28	−10	58	
18		IPCL	−55	−2	38	Inferior precentral lobule
19		SMA	−2	−2	55	Supplementary motor area
20		DLPFC_L	−40	39	30	Dorsolateral prefrontal cortex
21		DLPFC_R	38	41	26	
22		VOC_L	−47	−71	−8	Ventral occipital lobe
23		VOC_R	55	−64	−13	
